# Key concepts for clinical management of Molar Incisor Hypomineralization

**DOI:** 10.1590/1807-3107bor-2026.vol40suppl1061

**Published:** 2026-08-24

**Authors:** Aline de Almeida NEVES, Lourdes SANTOS-PINTO, Daniela RIOS, Vera Mendes SOVIERO

**Affiliations:** (a) Universidade Federal do Rio de Janeiro – UFRJ, School of Dentistry, Department of Orthodontics and Pediatric Dentistry, Rio de Janeiro, RJ, Brazil; (b) Universidade Estadual de São Paulo – Unesp, School of Dentistry, Department of Morphology and Pediatric Dentistry, Araraquara, SP, Brazil; (c) Universidade de São Paulo – USP, Bauru School of Dentistry, Department of Pediatric Dentistry, Orthodontics and Public Health, Bauru, SP, Brazil; (d) Universidade do Estado do Rio de Janeiro – UERJ, School of Dentistry, Department of Preventive and Community Dentistry, Rio de Janeiro, RJ, Brazil

**Keywords:** Molar Hypomineralization, Dental Enamel Hypomineralization, Developmental Defects of Enamel

## Abstract

Clinical management of patients with molar incisor hypomineralization (MIH) requires a comprehensive and multidimensional assessment that integrates clinical, behavioral, radiographic, functional, and psychosocial parameters. This article discusses key aspects of MIH management, including diagnosis and severity grading; non-invasive strategies aimed at preventing enamel breakdown; restorative management of severely affected molars, including the role of timed extractions; esthetic treatment to mask opacities in anterior teeth; and desensitizing approaches for the management of hypersensitivity. The literature for this narrative review was identified through targeted searches in PubMed for classic and recent publications on the clinical management of MIH. Reference lists of relevant articles were also hand-searched to identify additional relevant studies. Finally, future perspectives and emerging directions in MIH management are discussed.

## A holistic approach to the diagnosis and prognosis of affected teeth in children with MIH

Molar-incisor hypomineralization (MIH) is a developmental enamel defect that presents significant clinical challenges beyond the structural integrity of affected teeth. From an epidemiological perspective, MIH has emerged as a significant public health concern due to its high prevalence, worldwide distribution, and substantial impact on oral health and quality of life. MIH affects a considerable proportion of children globally, with a pooled global prevalence estimated at approximately 13.1%, and values that vary according to diagnostic criteria, age groups, and study populations.^1^

An accurate diagnosis and reliable prognosis require a comprehensive, multidimensional assessment that integrates clinical, behavioral, radiographic, functional, and psychosocial factors.^2^, ^3^ The diagnostic process begins with a detailed medical and dental history, including prenatal, perinatal, and early childhood conditions, as systemic disturbances during amelogenesis have been associated with the multifactorial etiology of MIH.^4^

The clinical examination should systematically evaluate morphological characteristics of enamel defects and radiographic assessment complements these findings by revealing pulpal involvement, hidden caries lesion and restoration defects. Moreover, MIH management should follow a holistic and child-centered approach, addressing not only the affected teeth but also the child’s functional, emotional, and behavioral needs. Treatment planning should consider factors such as pain perception, dental anxiety, treatment tolerance, and oral health-related quality of life.^4^ In addition, preventive and therapeutic strategies, including caries prevention, desensitization protocols, minimally invasive restorative interventions, and long-term follow-up, should be individualized according to each patient’s clinical characteristics and needs.^5^

Clinicians must also recognize the emotional and psychosocial burden associated with MIH. Children presenting visible anterior defects may experience reduced self-esteem, social embarrassment, and impaired social interactions, while chronic hypersensitivity may negatively affect eating habits, oral hygiene practices, sleep quality, and overall daily comfort.^6^ Moreover, children affected by MIH frequently experience hypersensitivity and repeated dental interventions, which may negatively influence cooperation and increase fear and anxiety during dental treatment.^7^ Addressing these concerns may improve treatment acceptance, adherence, and overall quality of life.

Therefore, contemporary treatment protocols to MIH management should integrate individualized clinical decision-making with strategies aimed at improving both oral health and patient well-being over time.

## Diagnosing, grading and staging severity

Since MIH was recognized and described as a specific condition,^2^ there has been a need to propose diagnostic criteria to identify and assess its severity in the clinic. The EAPD (European Academy of Paediatric Dentistry) criteria was proposed in 2003^8^ and reinforced in an updated policy document published in 2022.^5^ One advantage of the EAPD criteria is that they combine clinical aspects identified in the teeth with symptoms and concerns reported by the patient, resulting in diagnosis at the patient level. However, no color differentiation was suggested for teeth presenting only opacities. In addition, no distinction was made between post-eruptive enamel breakdown (PEB) restricted to enamel and PEB exposing dentin, nor between PEB occurring alone or in combination with caries lesion. In an attempt to overcome these limitations, the updated EAPD policy document^5^ suggested incorporating the grading systems proposed by Ghanim et al.^9^ to differentiate white-creamy from yellow-brownish opacities and to identify atypical caries lesions in teeth presenting PEB.

It has been extensively demonstrated that the darker the opacity, the lower the mineral and the higher the protein content of the enamel.^10^, ^11^, ^12^, ^13^, ^14^ Moreover, the validity of differentiating opacities in white-creamy and yellow brownish categories has been demonstrated in longitudinal clinical studies showing that darker opacities are at a higher risk of breakdown over time.^15^, ^16^, ^17^, ^18^, ^19^, ^20^ Therefore, when screening teeth for MIH, it is advisable to use a scoring system that grades not only the level of surface integrity (enamel or dentin), but also the color of the opacities not showing post-eruptive enamel breakdown.

Discriminating between PEB with or without caries lesion may be challenging. Not all PEB will necessarily evolve into caries lesion. A PEB exposing dentine, where plaque accumulation and soft dentine are observed, will be considered carious. According to the diagnostic criteria, caries lesions surrounded by hypomineralized enamel and/or involving tooth surfaces which are usually not affected by caries will be scored as atypical caries lesion.^9^

**Figure 1:**
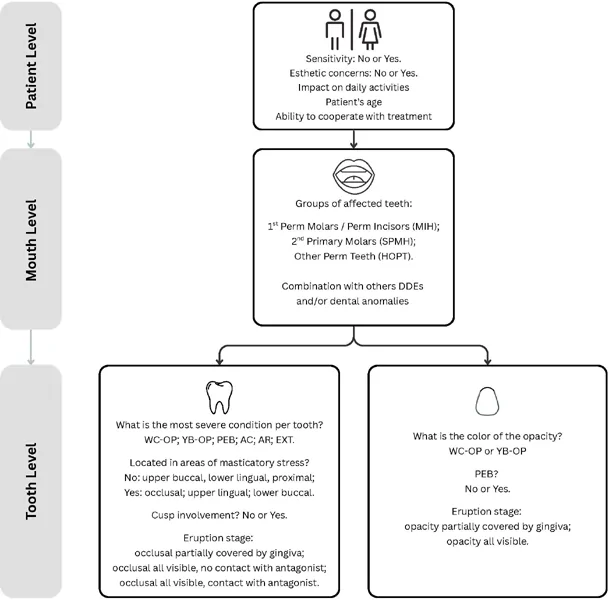
Flowchart recommending the assessment of clinical data at the patient level, mouth level, and tooth level during the examination of patients affected by MIH.

Figure 1 presents an examination flow, collecting clinical data from the patient level to the tooth surface level.

The association between MIH and caries deserves a comprehensive discussion. Several studies have reported that MIH patients present higher caries prevalence and higher caries index scores compared with those without MIH.^21^, ^22^, ^23^ As most of the studies are based on the DMF-T index, their results may overestimate the occurrence of caries because fillings are not necessarily placed due to caries, and enamel breakdown exposing dentin may be scored as cavitated caries even when no carious lesion is present. Therefore, the questions whether MIH increase the caries index or the need for restorative treatment is genuine in this context. Higher DMF-T values in MIH-patients reflect into a greater treatment need in permanent teeth but not necessarily caused solely by carious lesions.^24^, ^25^

It is also necessary to discuss whether MIH represents an indicator ofa higher caries risk at the patient or at the tooth level. Not only can post-eruptive enamel breakdown lead to dentine exposure, but the rough and porous enamel surface typically present in hypomineralized enamel also makes effective cleaning challenging and facilitates plaque accumulation. Moreover, the hypomineralized enamel may be more susceptible to caries development due to its lower mineral content and consequently reduced resistance to the demineralizing effects of acids from a cariogenic biofilm. Indeed, it has been shown that MIH-affected molars with compromised enamel and dentine exposure are more susceptible to faster caries progression under a cariogenic biofilm.^25^ These biological and clinical characteristics support the hypothesis that MIH-affected teeth may be more vulnerable to caries development than unaffected teeth.^26^ Nevertheless, the available evidence is still limited by methodological challenges, particularly the difficulty in distinguishing carious lesions from post-eruptive enamel breakdown and MIH-related restorative treatment when conventional caries indices are used. Consequently, further longitudinal studies employing more specific diagnostic criteria are required to clarify whether MIH is associated with an increased risk of caries development and whether this association occurs primarily at the tooth rather than the patient level.

## Key concepts for clinical management of MIH in molars

From a functional perspective, the clinical management of the first permanent molars is the most challenging aspect when treating patients with MIH. From non-invasive approaches aimed to prevent enamel breakdown to the management of severely affected molars, there is still limited evidence regarding the effectiveness of the available treatment options.

### Evidence of clinical efficacy of available treatment options to prevent PEB and caries lesions

Ideally, the treatment for permanent molars with demarcated opacities is the prevention of PEB. *In vitro* and *in vivo* studies have demonstrated that hypomineralized enamel exposed to remineralization agents (toothpastes containing casein phosphopeptide and amorphous calcium phosphate - CPP-ACP or hydroxyapatite, and fluoride varnish) shows improvement in mineral content, mineral density, physical strength, or a decrease in laser fluorescence values.^27^, ^28^ However, considering that MIH lesions are subsurface in nature, originating at the amelodentinal junction and often covered by an apparently intact superficial layer, these findings may also represent post-eruptive maturation or superficial remineralization rather than true recovery of the underlying defect. This is likely why there is no clinical evidence that any remineralization agent prevents enamel disintegration in the context of MIH. Although microscopic superficial modification of hypomineralized enamel occurs following exposure to remineralization agents, it does not necessarily mean that the treated enamel becomes less susceptible to PEB.^29^ Nonetheless, the recommendation for daily use of fluoridated toothpaste (≥ 1,000 ppm F) remains undisputed due to its well-documented benefits in the control of dental caries.

The effectiveness of fissure sealants in preventing and controlling occlusal caries lesion in permanent teeth is well stablished.^30^ Considering that MIH-affected molars are more prone to caries, when the patient is at caries risk, fissure sealants are among the recommended preventive strategies.^5^ While resin-based sealants show significantly higher retention rates,^31^, ^32^ the caries preventive effect is similar between GIC and resin-based sealants.^32^ However, the high retention rate of resin-based sealants depends on optimal moisture control, what is not achievable during early stages of tooth eruption. Therefore, GIC sealants are frequently used in MIH-affected molars, particularly when they are not fully erupted.^33^

As sealants act as a physical barrier by isolating the surface of pit and fissures from the biofilm accumulation, they protect MIH-affected molars against caries; however, the same protective effect has not been observed for PEB.^34^ This can be explained by the fact that demarcated opacities are not confined to the areas covered by the sealant. Even when the sealant is fully retained in the pit and fissure areas, PEB may occur in cusps or other tooth surfaces not directly protected by the sealant. It should also not be expected that fluoride released by the GIC will reinforce the surrounding hypomineralized enamel sufficiently to prevent PEB.^29^

All demarcated opacities may be prone to post-eruptive breakdown (PEB) and should be monitored; however, as yellow brownish opacities are at higher risk of PEB than creamy-white ones,^35^ it is recommended to monitor patients with darker opacities at shorter intervals. PEB detected at early stages can be covered and protected with GIC or resin-based materials, thereby postponing more invasive procedures.

### Evidence of clinical efficacy of available restorative treatment options for severe defects

The evidence to support restorative options for severely MIH-affected molars is still limited, primarily due to the considerable variability among the studies regarding study design, management of the surrounding hypomineralized enamel, criteria used to assess restoration success, and follow-up periods.^28^, ^36^

Nonetheless, with respect to direct restorations, composite resins have shown considerably higher success rates in medium- to long-term studies compared with glass ionomer cement (GIC) restorations.^28^, ^36^, ^37^ The success rate of GIC restorations has been reported to be as high as 86 to 98% in studies with follow-up limited to 18 months,^38^, ^39^, ^40^ but this drops to below 50% in studies with follow-up periods longer than 42 months.^37^, ^41^ In contrast, the success rate of composite resin restorations ranges from 70 to 74.5% after 42 months^42^ and reaches approximately 80% after 10 years.^41^ However, these findings should be interpreted with caution. Most conclusions regarding the superiority of composite resin over GIC are based on indirect comparisons across independent studies with different methodologies, follow-up periods, clinical settings, and outcome definitions. Direct evidence comparing both materials in MIH-affected teeth remains limited, with only a small number of randomized clinical trials available and inconsistent results reported.^43^, ^44^ Therefore, the current literature does not provide sufficient evidence to establish a clear consensus regarding the superiority of one material over the other. Further well-designed randomized clinical trials with long-term follow-up are needed to determine the most appropriate restorative material for MIH-affected teeth.

Across the studies, the influence of factors such as cavity size, cusp involvement, and stage of tooth eruption is often not considered in outcome analyses.^37^, ^39^, ^40^, ^45^, ^46^ Cusp involvement has been identified as the main reason for failure of composite resin restorations in both 12-month^47^ and 24-month follow-up studies.^48^

When indirect restorations are used, survival rates above 90% have been reported after three years or more, regardless of the material used (ceramic, resin, metal onlays or stainless-steel crowns - SSC).^36^, ^41^, ^42^, ^49^, ^50^, ^51^ Therefore, before indicating a direct restoration, it is advisable to assess whether PEB, in association or not with atypical caries, extends to multiple tooth surfaces and cusp areas. The higher failure rate of direct restorations compared with indirect restorations in MIH-affected molars may be related to inadequate indication of the restorative material or technique than the presence of hypomineralized enamel itself.^52^ One possible explanation for the superior survival rates reported for indirect restorations is that the hypomineralized enamel surrounding post-eruptive breakdown lesions is either removed during tooth preparation or subsequently protected by the restoration itself. This hypothesis is supported by recent findings showing that the longevity of composite restorations in MIH-affected teeth is significantly improved when residual hypomineralized enamel adjacent to the defect is removed before restoration placement compared with more conservative approaches that preserve such enamel.^36^ These findings suggest that restoration failure may be more closely related to the presence of unsupported or structurally compromised hypomineralized enamel at restoration margins than to the restorative material itself.^36^ However, the recommendation to routinely remove all remaining hypomineralized enamel should be interpreted with caution. In many MIH-affected teeth, demarcated opacities extend over large portions of the crown, and extensive removal of affected enamel may result in unnecessary loss of tooth structure, weakening of the remaining crown and earlier entry into the restorative cycle. Therefore, decisions regarding enamel removal should be individualized, balancing the potential benefits for restoration longevity against the principles of minimally invasive dentistry and long-term preservation of tooth structure.

In very young patients and in partially erupted molars, GIC may be indicated as a temporary protective filling until a long-term option can be provided.^40^ In such cases, when PEB extends to multiple surfaces, the use of an orthodontic band has been suggested to protect the GIC and enhance the longevity of the temporary filling.^53^

The highest survival rates have been reported for indirect laboratory-fabricated restorations.^28^, ^36^, ^37^, ^49^, ^50^ However, conventional indirect restorative procedures require more complex tooth preparation and a minimum stage of tooth eruption. In addition, they are more time-consuming and highly dependent on patient cooperation. Therefore, they are better indicated when the patient reaches adolescence or young adulthood.

Preformed SSC have also shown very high success rates^48^, ^54^, ^55^ and, more recently, have been proposed using a minimally invasive approach, without traditional tooth preparation.^48^, ^56^, ^57^ The use of SSC based on the Hall Technique principle,^58^ in which no tooth preparation is performed, has emerged as a promising strategy for severely affected molars, with PEB and/or atypical caries extending to multiple tooth surfaces and cusps. This approach is a non-invasive and practical procedure that may serve as either a temporary or longer-term protective restoration,^52^ although further clinical studies are still needed to support its widespread recommendation. Nevertheless, the application of this concept in MIH-affected molars may differ slightly from the original Hall Technique. In clinical practice, limited crown adjustments are frequently required to improve cervical adaptation and minimize occlusal discrepancies, allowing better fit and retention while still preserving the minimally invasive nature of the procedure.

**Figure 2:**
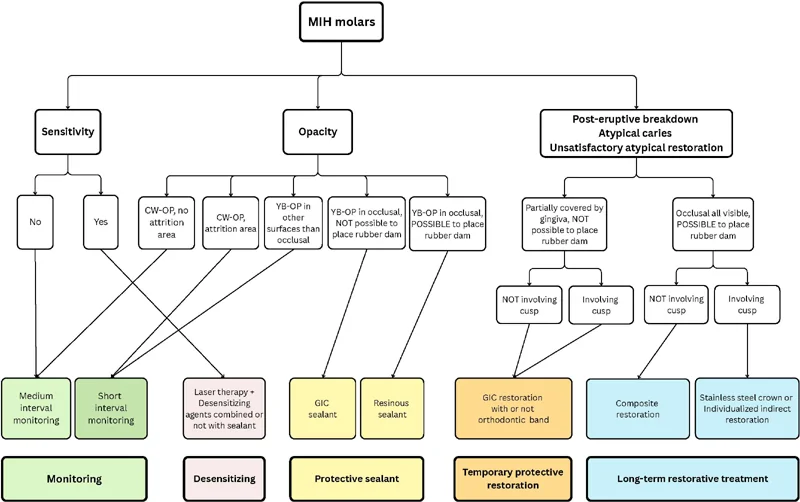
Treatment decision tree for MIH-affected molars. Monitoring, desensitizing approach, sealants and restorations are presented as treatment options.

Figure 2 presents a treatment decision tree for MIH-affected molars. Besides monitoring, desensitizing approach, sealants and restorations are among the treatment options.

### Evidence of clinical efficacy of timed extractions

Extraction may be the only option for severely affected molars where endodontic treatment and/or restoration is no longer feasible. Alternatively, extractions may be planned as a strategic approach for FPMs with poor medium- to long-term prognosis.^28^

The decision to extract severely affected FPM requires individual assessment and should take into account patient age, stage of development of the second molars, presence of third molars, as well as patient and parental expectations.^59^ In addition, the presence and type of malocclusion, facial pattern, space requirements, and overall orthodontic treatment objectives should be carefully evaluated, as these factors may substantially influence both the indication and timing of extraction.^60^ Therefore, treatment planning often requires close collaboration between pediatric dentists and orthodontists to ensure that the extraction decision is integrated into the patient’s overall occlusal and orthodontic management.

In patients without significant orthodontic concerns, extraction between 8 and 10 years ofage is generally considered the most favorable timing for spontaneous space closure.^60^ At this stage, the second permanent molar and premolar typically present early root development, usually with no more than one-third of the root formed, increasing the likelihood of favorable mesial migration and eruption of the second permanent molar into the position of the extracted FPM.^56^ However, when malocclusions are present, the optimal timing may differ from this recommendation and should be determined according to the individual orthodontic treatment plan. In some situations, delaying extraction may be advantageous, allowing the compromised FPM to be incorporated into a broader orthodontic strategy.^60^ Radiographic evaluation at this age may also provide useful information regarding the presence or absence of the developing third molar, which can contribute to long-term treatment planning. Moreover, a more favorable tooth migration is generally expected in the maxillary arch compared with the mandible.^59^, ^60^, ^61^, ^62^

Additionally, it is important to consider that extraction of FPM does not guarantee that the patient will have permanent molars free of enamel hypomineralization, because second permanent molars can also be affected by enamel hypomineralization.^52^, ^63^, ^64^, ^65^ Although they are typically less severely affected then FPM, they may still require restorative treatment and continuous monitoring.

## Key concepts for clinical management of MIH in anterior teeth

Patients with severe MIH often report difficulties in eating, drinking, and daily activities, such as toothbrushing. These challenges are reflected in higher scores for negative impact on oral symptoms and functional limitations in questionnaires assessing oral health related quality of life (OHRQoL).^66^ However, when visible opacities are present in anterior teeth, a negative impact on psychosocial domain may also be observed^67^. Treatments aimed to “mask” or “disguise” opacities in anterior teeth have been shown to significantly improve the socio-emotional wellbeing and the self-perception of tooth appearance in children and adolescents.^68^, ^69^ Therefore, patients who report concerns and negative impact on their quality of life due to affected anterior teeth should be considered eligible for esthetic interventions.^67^

Minimally invasive options, such as tooth bleaching, microabrasion, resin infiltration, or combinations of these approaches, should be preferred.^70^ Each option follows a different mechanism: bleaching aims to reduce the color difference between whitish opacities and the surrounding sound enamel and reduce the intensity of yellow-brown opacities; microabrasion removes superficial discolorations while resin infiltration restores the refractive index of the affected enamel by filling subsurface porosities.^71^

For effective masking with resin infiltration, adequate penetration of the resin into hypomineralized enamel is essential. Factors such as the presence and thickness of the pseudo normal surface layer and the depth of the lesion influence the level of resin penetration.^72^ In most cases of hypomineralization, the more porous enamel is covered by a hypermineralized surface layer, and thus, the lesion is wider in the subsurface than at the surface. Acid etching, combined or not with microabrasion, may provide access to the central part of the lesion but not to its margins.^73^ In fact, it has been demonstrated that resin infiltration is optimized by increasing both etching time and resin application time.^69^ However, even more effective results have been achieved through cycles of selective removal of superficial enamel using a polishing bur combined with short acid etching.^74^ Transillumination during the ethanol rewetting phase of the technique is a valuable tool for detecting residual closed lesion areas. In this situation, the bur should be selectively used again until a complete access to the lesion body is achieved.^74^

At-home bleaching has been demonstrated to convert yellowish opacities into whitish ones and to reduce the color contrast between the opacity and the surrounding sound enamel by the overall tooth whitening.^75^ Therefore, it has been strongly recommended as a first step prior to resin infiltration.^74^, ^75^

**Figure 3:**
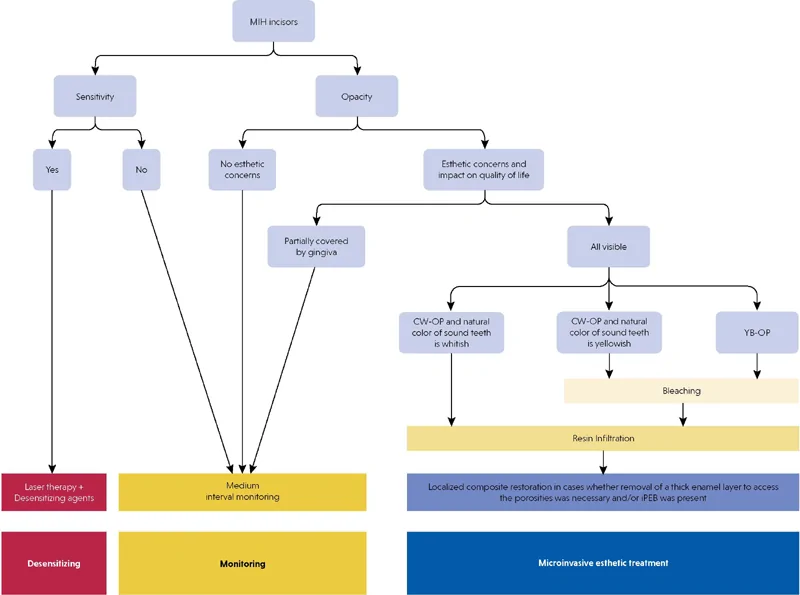
Treatment decision tree for MIH-affected incisors. Monitoring, desensitizing approach, and microinvasive esthetic treatment are presented as options.

In most cases, the microinvasive procedures are sufficient to achieve satisfactory masking of opacities. However, more complex cases may require restorative interventions. When removal of a thicker enamel layer is necessary to access deeper subsurface porosities, or when enamel breakdown is present, localized composite restorations may be require restoring the contour of the tooth surface.^70^, ^75^ Figure 3 presents a treatment decision tree for MIH-affected incisors. Besides monitoring, desensitizing approach, and microinvasive esthetic treatment are among the options.

## Key concepts for clinical management of hypersensitivity

Patients with MIH frequently report hypersensitivity during daily activities such as eating, toothbrushing, and inhalation of cold air.^7^ The prevalence of hypersensitivity ranges from 30 to 55% among patients with MIH, depending on the diagnostic method and the MIH severity.^76^ Although the exact mechanism leading to increased sensitivity in MIH-affected teeth is not fully understood, it appears to be related to the penetration of bacteria through enlarged interprismatic spaces, reaching the dentinal tubules and causing subclinical pulpal inflammation.^77^ Additionally, the highly porous enamel fails to adequately protect the underlying tissues from thermal, physical, and chemical external stimuli.^78^ In clinical practice, patients with MIH should be routinely asked about difficulties in daily activities such as consuming cold foods or drinks, toothbrushing, and chewing solid food.^78^

Occlusion of the enamel porosities is expected to relieve the symptoms associated to hypersensitivity. Desensitizing products, such as toothpastes containing 8% arginine and calcium carbonate,^79^ 10% microcrystalline hydroxyapatite,^80^ or silicon combined with calcium, and a CPP-ACP mousse,^81^ have been shown to significantly reduce sensitivity in patients with MIH, as measured by the Schiff Cold Air Sensitivity Scale (SCASS) following cold and tactile stimuli. Sealants have also demonstrated a significant effect in reducing hypersensitivity in MIH-affected molars regardless ofwhether resin-based or GIC materials are used.^82^, ^83^, ^84^ SCASS scores shown to decrease significantly after a single application of silver diamine fluoride (SDF)^85^, ^86^, ^87^ with additional benefit observed when a GIC sealant was placed over SDF.^85^, ^86^ The desensitizing effect of fluoride varnish applied weekly for four weeks has been shown to be potentialized by two sessions of photobiomo dulation using a low-power diode laser.^88^ Similarly, low-power laser therapy has been reported to enhance the effect of GIC sealants in reducing hypersensitivity in first permanent molars.^89^

A recommended approach to manage hypersensitivity involves a two-phase strategy: an initial phase using low-power laser therapy, followed by a second phase aimed at creating a physical barrier against external stimuli. Low-power laser therapy is thought to inhibit sensory nerve activity and promote the release of neurotransmitters, resulting in an analgesic effect, while desensitizing agents act by occluding enamel porosities and dentinal tubules.^78^

## Future perspectives

For many years, restorative failures in MIH-affected teeth have been primarily attributed to reduced adhesion to hypomineralized enamel, a concept largely derived from laboratory studies. 90 However, an important question remains unresolved: although bond strength to hypomineralized enamel may be reduced, is this reduction clinically sufficient to compromise restoration survival in vivo? This issue still requires further investigation. Based on the assumption that improved adhesion results in better restorative outcomes, some authors have advocated the removal of hypomineralized opacities surrounding PEB before restoration and reported higher survival rates following this approach.^37^ However, the benefits associated with opacity removal may not necessarily be explained by improved bonding alone. An alternative interpretation is that the residual hypomineralized enamel surrounding the restoration represents a structurally fragile substrate that remains susceptible to further post-eruptive breakdown. Consequently, what is often interpreted as restoration failure may, in some cases, reflect fracture of the surrounding enamel rather than loss of restoration retention itself. In this context, removal of the residual hypomineralized enamel may improve survival rates not only because it provides a more favorable bonding substrate, but also because it eliminates tissue that is inherently prone to future breakdown.

From this perspective, preserving and strengthening hypomineralized enamel rather than removing it may represent a more conservative and biologically oriented strategy. Current evidence suggests that conventional remineralizing agents and sealants are unable to substantially improve the mechanical properties of the defect.^33^, ^34^ Therefore, alternative approaches such as resin infiltration and enamel covering strategies have been proposed with the aim of reinforcing hypomineralized enamel and increasing its resistance to fracture.^91^ Future studies should determine whether these approaches are capable of effectively protecting MIH-affected enamel against post-eruptive breakdown. If proven effective, they could be implemented soon after tooth eruption to prevent hypomineralized enamel breakdown and reduce the future need for restorative intervention. In teeth that already present PEB, these strategies could also be combined with restorative procedures to protect residual hypomineralized enamel surrounding the restoration, thereby reducing the risk ofsubsequent enamel fracture. Additionally, these approaches may contribute to the management of hypersensitivity. By infiltrating or covering the porous hypomineralized enamel, these materials may reduce fluid movement and external stimuli transmission through the defect while simultaneously reinforcing the weakened enamel structure. Consequently, they have the potential to provide a dual benefit by both increasing resistance to post-eruptive breakdown and alleviating one of the most common and clinically relevant symptoms associated with MIH.

In addition to these considerations, another important aspect concerns to the current nomenclature of MIH, which may no longer fully reflect the true extent and clinical variability of the condition. Although the term implies that the defect is limited to first permanent molars and permanent incisors, a growing body of evidence demonstrates that hypomineralization may also affect second permanent molars, premolars, permanent canines, and primary teeth, particularly second primary molars and primary canines. Furthermore, numerous studies indicate that MIH and hypomineralized second primary molars (HSPM) are not distinct disorders but rather different manifestations within a broader spectrum of developmental enamel hypomineralization. Collectively, these findings suggest that the current terminology may be overly restrictive and no longer fully representative of the clinical reality. Consequently, it may be timely to reconsider the nomenclature and adopt a more comprehensive and inclusive term that better reflects the diversity of affected teeth, improves diagnostic consistency, and facilitates the development of preventive and therapeutic strategies across the full spectrum of enamel hypomineralization defects.^92^

## Data Availability

The authors declare that all data generated or analyzed during this study are included in this published article.

## References

[1] Schwendicke F, Elhennawy K, Reda S, Bekes K, Manton DJ, Krois J. (2018). Global burden of molar incisor hypomineralization. J Dent.

[2] Weerheijm KL, Jälevik B, Alaluusua S. (2001). Molar-incisor hypomineralisation. Caries Res.

[3] Elhennawy K, Schwendicke F. (2016). Managing molar-incisor hypomineralization: a systematic review. J Dent.

[4] Juárez-López ML, Salazar-Treto LV, Hernández-Monjaraz B, Molina-Frechero N. (2023). Etiological factors of molar incisor hypomineralization: a systematic review and meta-analysis. Dent J.

[5] Lygidakis NA, Garot E, Somani C, Taylor GD, Rouas P, Wong FS. (2022). Best clinical practice guidance for clinicians dealing with children presenting with molar-incisor-hypomineralisation (MIH): an updated European Academy of Paediatric Dentistry policy document. Eur Arch Paediatr Dent.

[6] Shields S, Chen T, Crombie F, Manton DJ, Silva M. (2024). The impact of molar incisor hypomineralisation on children and adolescents: a narrative review. Healthcare (Basel).

[7] Jalevik B, Klingberg GA. (2002). Dental treatment, dental fear and behaviour management problems in children with severe enamel hypomineralization of their permanent first molars. Int J Paediatr Dent.

[8] Weerheijm KL, Duggal M, Mejàre I, Papagiannoulis L, Koch G, Martens LC, (2003). Judgement criteria for molar incisor hypomineralisation (MIH) in epidemiologic studies: a summary of the European meeting on MIH held in Athens, 2003. European Acad Paediatr Dent.

[9] Ghanim A, Elfrink M, Weerheijm K, Marino R, Manton D. (2015). A practical method for use in epidemiological studies on enamel hypomineralisation. Eur Arch Paediatr Dent.

[10] Bozal CB, Kaplan A, Ortolani A, Cortese SG, Biondi AM. (2015). Ultrastructure of the surface of dental enamel with molar incisor hypomineralization (MIH) with and without acid etching. Acta Odontol Latinoam.

[11] Fearne J, Anderson P, Davis GR 3rd. (2004). 3D X-ray microscopic study of the extent of variations in enamel density in first permanent molars with idiopathic enamel hypomineralisation. Br Dent J.

[12] Farah R, Drummond B, Swain M, Williams S. (2010). Linking the clinical presentation of molar-incisor hypomineralisation to its mineral density. Int J Paediatr Dent.

[13] Farah RA, Monk BC, Swain MV, Drummond BK. (2010). Protein content of molar-incisor hypomineralisation enamel. J Dent.

[14] Elhennawy K, Manton DJ, Crombie F, Zaslansky P, Radlanski RJ, Jost-Brinkmann PG, (2017). Structural, mechanical and chemical evaluation of molar-incisor hypomineralization-affected enamel: a systematic review. Arch Oral Biol.

[15] Cabral RN, Nyvad B, Soviero VL, Freitas E, Leal SC. (2020). Reliability and validity of a new classification of MIH based on severity. Clin Oral Investig.

[16] Neves AB, Americano GC, Soares DV, Soviero VM. (2019). Breakdown of demarcated opacities related to molar-incisor hypomineralization: a longitudinal study. Clin Oral Investig.

[17] Costa-Silva CM, Ambrosano GM, Jeremias F, Souza JF, Mialhe FL. (2011). Increase in severity of molar-incisor hypomineralization and its relationship with the colour of enamel opacity: a prospective cohort study. Int J Paediatr Dent.

[18] Arslanagić A, Marković N, Bajrić E, Burnazović Ristića L. (2020). Demarcated opacities as predictors of progression of the molar incisor hypomineralisation: a pilot study. Acta Stomatol Croat.

[19] Bullio Fragelli CM, Jeremias F, Souza JF, Paschoal MA, Cordeiro RCL, Santos-Pinto L. (2015). Longitudinal Evaluation of the structural integrity of teeth affected by molar incisor hypomineralisation. Caries Res.

[20] Jorge RC, GorniReis PP, Marañn-Vásquez GA, Masterson D, Maia LC, Mendes Soviero V. (2022). Are yellow-brownish opacities in hypomineralized teeth more prone to breakage than white-creamy ones? A systematic review. Clin Oral Investig.

[21] Americano GC, Jacobsen PE, Soviero VM, Haubek D. (2017). A systematic review on the association between molar incisor hypomineralization and dental caries. Int J Paediatr Dent.

[22] Gevert MV, Wambier LM, Ito LY, Souza JF, Chibinski AC. (2024). Which are the clinical consequences of Molar Incisor hypomineralization (MIH) in children and adolescents? Systematic review and meta-analysis. Clin Oral Investig.

[23] Villani FA, Aiuto R, Dioguardi M, Paglia L, Caruso S, Gatto R, (2023). Caries prevalence and molar incisor hypomineralisation (MIH) in children. Is there an association? A systematic review. Eur J Paediatr Dent.

[24] Americano GC, Jorge RC, Moliterno LF, Soviero VM. (2016). Relating molar incisor hypomineralization and caries experience using the decayed, missing, or filled index. Pediatr Dent.

[25] Mendonça FL, Martins DD, Grizzo IC, Masson LA, Di Campli FG, Souza IM, (2024). What can impact on the presence of carious lesions in first permanent molars? Revisiting the association between MIH and caries. Clin Oral Investig.

[26] Garot E, Vieira AR, Manton DJ. (2024). Molar incisor hypomineralisation as a dental caries risk factor - is it true?. Monogr Oral Sci.

[27] Enax J, Amaechi BT, Farah R, Liu JA, Schulze Zur Wiesche E, Meyer F. (2023). Remineralization Strategies for Teeth with Molar Incisor Hypomineralization (MIH): A Literature Review. Dent J.

[28] Somani C, Taylor GD, Garot E, Rouas P, Lygidakis NA, Wong FS. (2022). An update of treatment modalities in children and adolescents with teeth affected by molar incisor hypomineralisation (MIH): a systematic review. Eur Arch Paediatr Dent.

[29] Buzalaf MA, Rios D. (2024). Is it possible to remineralise hypomineralised enamel lesions in patients with molar incisor hypomineralisation?. Monogr Oral Sci.

[30] Ahovuo-Saloranta A, Forss H, Walsh T, Nordblad A, MäeläM, Worthington HV. (2017). Pit and fissure sealants for preventing dental decay in permanent teeth. Cochrane Database Syst Rev.

[31] Kühnisch J, Mansmann U, Heinrich-Weltzien R, Hickel R. (2012). Longevity of materials for pit and fissure sealing: results from a meta-analysis. Dent Mater.

[32] Alirezaei M, Bagherian A, Sarraf Shirazi A. (2018). Glass ionomer cements as fissure sealing materials: yes or no?: A systematic review and meta-analysis. J Am Dent Assoc.

[33] Bekes K. (2024). Fissure sealants for managing hypersensitivity and posteruptive breakdown in mih-affected molars. Monogr Oral Sci.

[34] Schraverus MS, Olegário IC, Bonifácio CC, González AP, Pedroza M, Hesse D. (2021). Glass ionomer sealants can prevent dental caries but cannot prevent posteruptive breakdown on molars affected by molar incisor hypomineralization: one-year results of a randomized clinical trial. Caries Res.

[35] Jorge RC, Reis PP, Soviero VM. (2024). Understanding the enamel disintegration and posteruptive breakdown of demarcated opacities on molar incisor hypomineralisation. Monogr Oral Sci.

[36] Lopes-Fatturi A, Wambier L, Rolim TZ, Reis A, Souza JF. (2022). Restorative techniques for permanent first molars affected by hypomineralization: a systematic review. Pediatr Dent.

[37] Linner T, Khazaei Y, Bücher K, Pfisterer J, Hickel R, Kühnisch J. (2020). Comparison of four different treatment strategies in teeth with molar-incisor hypomineralization-related enamel breakdown: a retrospective cohort study. Int J Paediatr Dent.

[38] Grossi JA, Cabral RN, Ribeiro AP, Leal SC. (2018). Glass hybrid restorations as an alternative for restoring hypomineralized molars in the ART model. BMC Oral Health.

[39] Gatón-Hernandéz P, Serrano CR, Silva LA, Castañeda ER, Silva RA, Pucinelli CM, (2020). Minimally interventive restorative care of teeth with molar incisor hypomineralization and open apex-A 24-month longitudinal study. Int J Paediatr Dent.

[40] Fragelli CM, Souza JF, Jeremias F, Cordeiro RC, Santos-Pinto L. (2015). Molar incisor hypomineralization (MIH): conservative treatment management to restore affected teeth. Braz Oral Res.

[41] Mejàre I, Bergman E, Grindefjord M. (2005). Hypomineralized molars and incisors of unknown origin: treatment outcome at age 18 years. Int J Paediatr Dent.

[42] Kotsanos N, Kaklamanos EG, Arapostathis K. (2005). Treatment management of first permanent molars in children with Molar-Incisor Hypomineralisation. Eur J Paediatr Dent.

[43] Kaya R, Yavuz BS, Dokumacigil NK, Kargül B. (2023). A randomized clinical trial of short fiber reinforced composite and glass hybrid restoration for molars affected by molar hypomineralization. Pediatr Dent.

[44] Ozsoy M, Erken Gungor O. (2024). Management of severity lesions of hypomineralized molars (MIH) with different treatment alternatives: 9-month results of a clinical trial. J Clin Pediatr Dent.

[45] Sõnmez H, Saat S. (2017). A clinical evaluation of deproteinization and different cavity designs on resin restoration performance in MIH-affected molars: two-year results. J Clin Pediatr Dent.

[46] Durmus B, Sezer B, Tugcu N, Caliskan C, Bekiroglu N, Kargul B. (2021). Two-year survival of high-viscosity glass ionomer in children with molar incisor hypomineralization. Med Princ Pract.

[47] Rolim TZ, Costa TR, Wambier LM, Chibinski AC, Wambier DS, Assunção LRS, (2021). Adhesive restoration of molars affected by molar incisor hypomineralization: a randomized clinical trial. Clin Oral Investig.

[48] Farias AL, Rojas-Gualdrón DF, Mejía JD, Bussaneli DG, Santos-Pinto L, Restrepo M. (2022). Survival of stainless-steel crowns and composite resin restorations in molars affected by molar-incisor hypomineralization (MIH). Int J Paediatr Dent.

[49] Gaardmand E, Poulsen S, Haubek D. (2013). Pilot study of minimally invasive cast adhesive copings for early restoration of hypomineralised first permanent molars with post-eruptive breakdown. Eur Arch Paediatr Dent.

[50] Dhareula A, Goyal A, Gauba K, Bhatia SK, Kapur A, Bhandari S. (2019). A clinical and radiographic investigation comparing the efficacy of cast metal and indirect resin onlays in rehabilitation of permanent first molars affected with severe molar incisor hypomineralisation (MIH): a 36-month randomised controlled clinical trial. Eur Arch Paediatr Dent.

[51] Koch MJ, García-Godoy F. (2000). The clinical performance of laboratory-fabricated crowns placed on first permanent molars with developmental defects. J Am Dent Assoc.

[52] Guerra BM, Jorge RC, Reis PP, Machado GF, Fidalgo TK, Soviero VM. (2025). Prevalence of molar incisor hypomineralization and demands for treatment according to the severity of its clinical manifestation. Clin Oral Investig.

[53] Ahmed AT, Kratunova E, Dean JA, Wright T, Khader Y, Alrayyes S. (2026). Perspectives of US pediatric dentistry faculty on the clinical management of molar hypomineralization. J Am Dent Assoc.

[54] Geduk N, Ozdemir M, Erbas Unverdi G, Ballikaya E, Cehreli ZC. (2023). Clinical and radiographic performance of preformed zirconia crowns and stainless-steel crowns in permanent first molars: 18-month results of a prospective, randomized trial. BMC Oral Health.

[55] Talekar AL, Waggoner WF, Silotry TM, Musale PK, Chaudhari GS. (2023). Prospective, randomized, clinical evaluation of preformed zirconia crowns and stainless steel crowns on permanent first molars: 12-month results. Pediatr Dent.

[56] Grizzo IC, Bisaia A, Di Leone CC, Di Campli FG, Mendonça FL, Rios D. (2022). Revisiting the use of stainless steel crowns for treatment of molar-incisor hypomineralization: a case series. Gen Dent.

[57] Guerra BM, Reis PP, Jorge RC, Soviero VM. (2021). Adaptação da Hall Technique para o tratamento restaurador de molares permanentes hipomineralizados: uma série de casos. Rev Assoc Paul Cir Dent.

[58] Innes N, Evans D, Stewart M, Keightley A. (2015). The hall technique: a minimal intervention and child friendly approach to managing the carious primary molar. University of Dundee.

[59] Ashley P, Noar J. (2019). Interceptive extractions for first permanent molars: a clinical protocol. Br Dent J.

[60] Garib D, Rios D, Ciantelli TL, Miranda F. (2024). Orthodontic analysis behind the extraction of hypomineralised first molars. Monogr Oral Sci.

[61] Stabilini SA, Giannini L, Cenzato N, Olivi Mocenigo F, Salerno C, Maspero C, (2025). Spontaneous second molar migration after mih-affected first molars extraction: a radiographic-based method evaluation. Children (Basel).

[62] Masri AA, Mourad MS, Splieth CH, Krey KF, Schmoeckel J. (2025). Extraction of first permanent molars in children-a comprehensive review of history, aim, Space Closure and Other Consequences. J Clin Med.

[63] Kevrekidou A, Kosma I, Kotsanos I, Arapostathis KN, Kotsanos N. (2021). Enamel opacities in all other than Molar Incisor Hypomineralisation index teeth of adolescents. Int J Paediatr Dent.

[64] Hanan SA, Farias AL, Santos-Pinto L. (2023). Molar Incisor Hypomineralization in adolescents and adults and its association with facial profile and occlusion. Clin Oral Investig.

[65] Farias AL, Rojas-Gualdrón DF, Bussaneli DG, Santos-Pinto L, Mejía JD, Restrepo M. (2022). Does molar-incisor hypomineralization (MIH) affect only permanent first molars and incisors? New observations on permanent second molars. Int J Paediatr Dent.

[66] Jalevik B, Sabel N, Robertson A. (2022). Can molar incisor hypomineralization cause dental fear and anxiety or influence the oral health-related quality of life in children and adolescents?-a systematic review. Eur Arch Paediatr Dent.

[67] Lima MD, Moura LF, Paiva SM, Lima CC, Moura MS, Paschoal MA. (2024). Impact of molar incisor hypomineralisation on quality of life. Monogr Oral Sci.

[68] Hasmun N, Lawson J, Vettore MV, Elcock C, Zaitoun H, Rodd H. (2018). Change in oral health-related quality of life following minimally invasive aesthetic treatment for children with molar incisor hypomineralisation: a prospective study. Dent J.

[69] Athayde GD, Reis PP, Jorge RC, Americano GC, Fidalgo TK, Soviero VM. (2022). Impact of masking hypomineralization opacities in anterior teeth on the esthetic perception of children and parents: a randomized controlled clinical trial. J Dent.

[70] Silva JP, Mendonça RP, Rocha RS, Rios D, Bresciani E. (2024). Re-establishing the esthetics of incisors in patients with molar incisor hypomineralisation. Monogr Oral Sci.

[71] Sara Mistry N, Muwaquet Rodriguez S. (2024). Comparison of aesthetic treatments for molar-incisor hypomineralisation: systematic review and meta-analysis. Saudi Dent J.

[72] Paris S, Soviero VM, Schuch M, Meyer-Lueckel H. (2013). Pretreatment of natural caries lesions affects penetration depth of infiltrants in vitro. Clin Oral Investig.

[73] Denis M, Atlan A, Vennat E, Tirlet G, Attal JP. (2013). White defects on enamel: diagnosis and anatomopathology: two essential factors for proper treatment (part 1). Int Orthod.

[74] Torres CR, Pereira TP, Effenberger S, Borges AB. (2025). Optimizing resin infiltration procedure in molar incisor hypomineralization lesions. J Esthet Restor Dent.

[75] Greenwall L, Cebula M, Greenwall-Cohen J, Schwendicke F, Effenberger S. (2026). To bleach or not to bleach?-the role of bleaching in the clinical workflow for the treatment of demarcated opacities in anterior teeth. J Esthet Restor Dent.

[76] Santos PS, Vitali FC, Fonseca-Souza G, Maia LC, Cardoso M, Feltrin-Souza J, (2024). Dentin hypersensitivity and toothache among patients diagnosed with Molar-Incisor Hypomineralization: a systematic review and meta-analysis. J Dent.

[77] Rodd HD, Boissonade FM, Day PF. (2007). Pulpal status of hypomineralized permanent molars. Pediatr Dent.

[78] Paschoal MA, Carvalho FK, Lima MD, Leal SC. (2024). Factors associated with hypersensitivity, management, and treatment options for teeth with molar incisor hypomineralisation. Monogr Oral Sci.

[79] Bekes K, Heinzelmann K, Lettner S, Schaller HG. (2017). Efficacy of desensitizing products containing 8% arginine and calcium carbonate for hypersensitivity relief in MIH-affected molars: an 8-week clinical study. Clin Oral Investig.

[80] Ehlers V, Reuter AK, Kehl EB, Enax J, Meyer F, Schlecht J, (2021). Efficacy of a toothpaste based on microcrystalline hydroxyapatite on children with hypersensitivity caused by MIH: a randomised controlled trial. Oral Health Prev Dent.

[81] Pasini M, Giuca MR, Scatena M, Gatto R, Caruso S. (2018). Molar incisor hypomineralization treatment with casein phosphopeptide and amorphous calcium phosphate in children. Minerva Stomatol.

[82] Bekes K, Amend S, Priller J, Zamek C, Stamm T, Kramer N. (2022). Hypersensitivity relief of MIH-affected molars using two sealing techniques: a 12-week follow-up. Clin Oral Investig.

[83] Karim R, Baider M, Splieth CH, Schmoeckel J. (2025). Efficiency of glass ionomer sealant application in reducing hypersensitivity in MIH-molars in schoolchildren immediately and after 12 weeks. Eur Arch Paediatr Dent.

[84] Baraka M, Elwardani G, Hamed H, Zeitoun SI. (2025). Management of hypersensitivity in molar incisor hypomineralisation using photobiomodulation and sealants in children: a randomized clinical trial. J Evid Based Dent Pract.

[85] Erbas Unverdi G, Ballikaya E, Cehreli ZC. (2024). Clinical comparison of silver diamine fluoride (SDF) or silver-modified atraumatic restorative technique (SMART) on hypomineralised permanent molars with initial carious lesions: 3-year results of a prospective, randomised trial. J Dent.

[86] Ballikaya E, Ünverdi GE, Cehreli ZC. (2022). Management of initial carious lesions of hypomineralized molars (MIH) with silver diamine fluoride or silver-modified atraumatic restorative treatment (SMART): 1-year results of a prospective, randomized clinical trial. Clin Oral Investig.

[87] Ahmed WB, Masri AA, Splieth CH, Schmoeckel J. (2025). Silver fluoride/potassium iodide application in hypersensitive MIH-affected molars in schoolchildren: prospective clinical evaluation of immediate and mid-term effects in desensitization. Quintessence Int (Berl, Ger : 1985).

[88] Muniz RS, Carvalho CN, Aranha AC, Dias FM, Ferreira MC. (2020). Efficacy of low-level laser therapy associated with fluoride therapy for the desensitisation of molar-incisor hypomineralisation: randomised clinical trial. Int J Paediatr Dent.

[89] Fossati AL, Mandetta AR, Sobral AP, Bruno LH, Viarengo NO, Sertaje MR, (2025). Randomized clinical trial of photobiomodulation and glass ionomer sealant for hypersensitivity in molar incisor hypomineralization. Sci Rep.

[90] Wang L, Mosquim V, Zabeu GS, Feltrin-Souza J. (2024). MIH-affected substrate: an overview of adhesive challenges to the dental structures. Monogr Oral Sci.

[91] Marouane O, Nefzaoui M, Manton DJ, Cebula M, Schwendicke F, Effenberger S. (2026). Transillumination-aided infiltration of mih-affected molars: evaluation of the clinical workflow. Int J Paediatr Dent.

[92] Lopes LB, Garot E, Elfrink M, Sabel N, Martins IC, Declerck D. (2026). Molar incisor hypomineralisation (MIH) and hypomineralised second primary molars (HSPM): do we need to alter terminology and definition? A scoping review. Eur Arch Paediatr Dent.

